# HIPK2 deficiency causes chromosomal instability by cytokinesis failure and increases tumorigenicity

**DOI:** 10.18632/oncotarget.3583

**Published:** 2015-03-14

**Authors:** Davide Valente, Gianluca Bossi, Alice Moncada, Mara Tornincasa, Stefania Indelicato, Salvatore Piscuoglio, Eva Diamantis Karamitopoulou, Armando Bartolazzi, Giovanna Maria Pierantoni, Alfredo Fusco, Silvia Soddu, Cinzia Rinaldo

**Affiliations:** ^1^ Experimental Oncology Laboratory, Regina Elena National Cancer Institute, Rome, Italy; ^2^ Laboratory of Medical Physics and Expert Systems, Regina Elena National Cancer Institute, Rome, Italy; ^3^ Department of Molecular Medicine and Medical Biotechnology, Federico II University, Naples, Italy; ^4^ Pathology Research Laboratory, Sant'Andrea University Hospital, Rome, Italy; ^5^ Institute of Pathology, University Hospital of Basel, Basel, Switzerland; ^6^ Translational Research, Institute of Pathology, University of Bern, Bern, Switzerland; ^7^ Institute of Molecular Biology and Pathology (IBPM), National Research Council of Italy (CNR), c/o Sapienza University, Rome, Italy; ^8^ Present address: Institute of Medical Genetics, Catholic University, Rome, Italy; ^9^ Present address: Department of Pathology, Memorial Sloan-Kettering Cancer Center, New York, USA

**Keywords:** HIPK2, cytokinesis failure, near-tetraploidy, CIN, tumorigenicity

## Abstract

HIPK2, a cell fate decision kinase inactivated in several human cancers, is thought to exert its oncosuppressing activity through its p53-dependent and -independent apoptotic function. However, a HIPK2 role in cell proliferation has also been described. In particular, HIPK2 is required to complete cytokinesis and impaired HIPK2 expression results in cytokinesis failure and tetraploidization. Since tetraploidy may yield to aneuploidy and chromosomal instability (CIN), we asked whether unscheduled tetraploidy caused by loss of HIPK2 might contribute to tumorigenicity. Here, we show that, compared to Hipk2+/+ mouse embryo fibroblasts (MEFs), hipk2-null MEFs accumulate subtetraploid karyotypes and develop CIN. Accumulation of these defects inhibits proliferation and spontaneous immortalization of primary MEFs whereas increases tumorigenicity when MEFs are transformed by E1A and Harvey-Ras oncogenes. Upon mouse injection, E1A/Ras-transformed hipk2-null MEFs generate tumors with genetic alterations resembling those of human cancers derived by initial tetraploidization events, such as pancreatic adenocarcinoma. Thus, we evaluated HIPK2 expression in different stages of pancreatic transformation. Importantly, we found a significant correlation among reduced HIPK2 expression, high grade of malignancy, and high nuclear size, a marker of increased ploidy. Overall, these results indicate that HIPK2 acts as a caretaker gene, whose inactivation increases tumorigenicity and causes CIN by cytokinesis failure.

## INTRODUCTION

HIPK2 (Homeodomain-Interacting Protein Kinase 2) is an evolutionary conserved kinase that acts in a variety of signaling pathways including p53-dependent and -independent apoptosis, differentiation, angiogenesis, antiviral response, and transcriptional regulation [1,2] Inactivation of HIPK2 through different mechanisms is present in several human tumors [1,3] and *Hipk2−/−* and *+/−* mice are more prone than *Hipk2+/+* mice in developing tumors by classical two-stage skin carcinogenesis or following ionizing radiation [4, 5]. Reducing HIPK2 expression by RNA interference impairs apoptosis and induces resistance to different anticancer treatments [6,7], supporting the common idea that HIPK2 oncosuppressing activity resides in its pro-apoptotoic function. In apparent contrast, inactivation of HIPK2 was also shown to impair proliferation *in vitro* and *in vivo*, in different cell types including sensory neurons, MEFs, bone marrow, and fetal liver cells [8-12], suggesting that HIPK2 also acts as a pro-proliferative factor. Indeed, we have recently demonstrated that HIPK2 localizes at the midbody and regulates cytokinesis, the final step of the cell cycle and that HIPK2 depletion results in cytokinesis failure and binucleation in human and mouse cells in vitro and in adult *Hipk2−/−* mouse liver [13]. In particular, we have shown that HIPK2 controls cytokinesis by phosphorylating the histone H2B at serine 14 at the midbody [13].

Proper cytokinesis is essential for maintaining ploidy and genome stability. Cytokinesis failure may indirectly generate aneuploidy and CIN because binucleated tetraploid cells can progressively lose or gain chromosomes during aberrant rounds of mitosis. The consequent de-polyploidization cascade ultimately results in near-tetraploid karyotypes [14-18]. Several factors including p53, pRb, LATS2 and some of their functional partners can favor or inhibit the survival of tetraploid cells, promote CIN and induce CIN tolerance mechanisms [18-24]. Thus, aneuploidy and CIN can either promote or inhibit tumor formation depending on the extent of the CIN, the type of affected tissues and their genetic background [25,26]. In human cancer, tetraploidy is present in the early and intermediate stages of different developing tumors, such as pancreas, colorectal, mammary, esophageal, and cervical cancers and it is thought to be responsible for the emergence of aneuploid karyotypes with high chromosome numbers [18, 27-28].

Pancreatic ductal adenocarcinomas arise from precursor lesions after a series of molecular alterations that correspond to distinct histopathological entities. These non-invasive lesions are termed pancreatic intraepithelial neoplasia (PanIN) and are separated into three grades. Failure of cytokinesis is considered as a major mechanism underlying tetraploidization and centrosome amplification in this type of cancer. Indeed, cytokinesis failure and the tendency of tetraploid cells to evade the tetraploidy checkpoint are frequently observed in an acinarductal transdifferentiating culture model of pancreatic carcinogenesis, predisposing to pleiotropic mitotic defects [29]. Recent studies have identified molecular alterations that occur in PanIn as they progress to invasive ductal adenocarcinoma [30-32]. However, the molecular mechanisms and genes involved in the cytokinesis failure in this model of tumor progression are still unknown.

Here, we examined whether cytokinesis failure caused by loss of hipk2 can generate aneuploidy and CIN and whether they might contribute to the pro-tumorigenic role played by hipk2 deficiency. We observed that in E1A/Ras-transformed MEFs, the absence of hipk2 leads to aneuploidy and CIN that associate with increased tumorigenicity and formation of highly aggressive tumors with sub-tetraploid karyotypes. Of relevance, hipk2 absence leads to aneuploidy and CIN also in primary MEFs. However, in this non-transformed context, the absence of Hipk2 alone is not sufficient to promote tolerance to karyotype defects and hipk2-null MEFs stop proliferating in a p53-dependent manner. Altogether, these results indicate that the aneuploidy and CIN induced by hipk2 absence play an important role mainly in tumor progression rather than in tumor promotion. Consistently, we found a progressive reduction of HIPK2 expression in the increasingly malignant stages of pancreatic adenocarcinomas.

## RESULTS

### E1A/Ras-transformed *Hipk2−/−* MEFs show higher rates of cytokinesis failure than E1A/Ras *Hipk2+/+* MEFs

**Figure 1:**
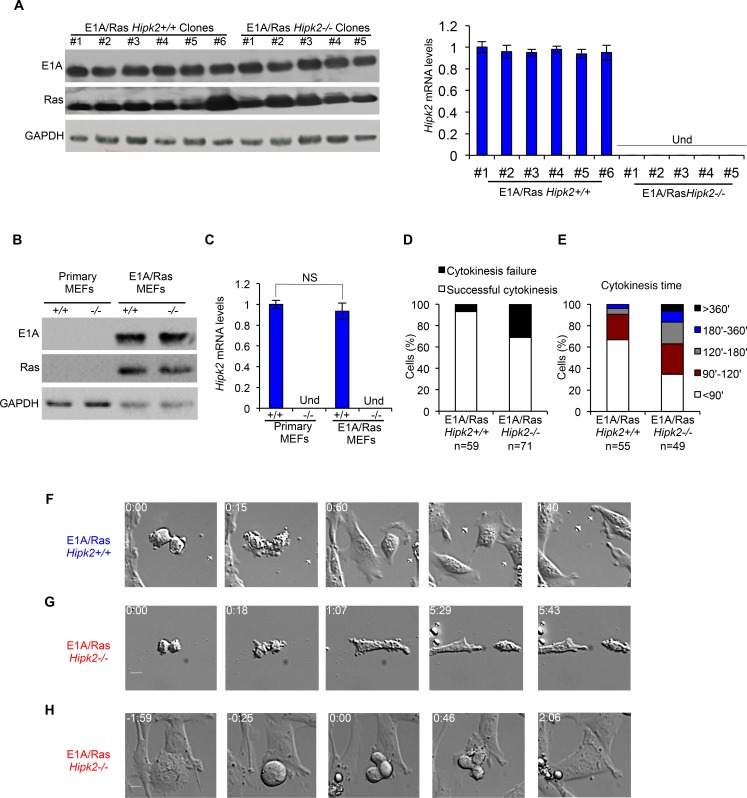
E1A/Ras *Hipk2−/−* MEFs show cytokinesis defects A-C, Stable E1A/Ras expressing clones and polyclonal populations were analyzed by WB and by real time RT-PCR. Representative WB for indicated proteins are shown (A left, and B), GAPDH expression was used as loading control. Hipk2 mRNA relative fold-enrichments were determined by the 2^−ΔΔCt^ method, using Actin as normalizer, and representative graphs are shown (A right, and C); data are represented as mean ± Standard Deviation (SD). NS, not significant. Und, undeterminable since no specific *Hipk2* mRNA amplification occurs, as expected in hipk2 null cells. D-H, Asynchronous E1A/Ras *Hipk2+/+* and *−/−* MEFs were analyzed by video time lapse microscopy at passage 3 after the establishment of stable expressing E1A/Ras polyclonal populations. D, The percentage of mononucleated cells with the indicated outcome is reported. E, Cytokinesis time was evaluated for each mononucleated cell successfully completing the cell division and the percentage of cells with the indicated cytokinesis time is reported. F-H, Still images related to Supplementary Movies S1, S2, and S3 are shown in F, G, and H, respectively.

To evaluate whether cytokinesis failure and tetraploidization caused by hipk2 inactivation can generate aneuploidy and CIN, we transformed early-passage primary *Hipk2*+/+ and −/− MEFs derived from the same littermate, by stably expressing the *E1A* and *Harvey-Ras* oncogenes. The expression levels of the two oncogenes and the *Hipk2* mRNA levels were assessed on single-cell clones (Figure 1A) and polyclonal populations stably expressing E1A and Ras (E1A/Ras MEFs) (Figures 1B-C). An initial characterization of these cells showed that, relative to their transfection efficiency, the *Hipk2−/−* MEFs yield a reproducible higher number of E1A/Ras-expressing colonies compared with the *Hipk2+/+* counterparts (Supplementary Figure S1A), suggesting that hipk2 absence might facilitate transformation, at least in these conditions.

Next, we verified whether Hipk2 absence leads to cytokinesis failure in the transformed MEFs, as we previously observed in other conditions [13]. Polyclonal populations of E1A/Ras *Hipk2*+/+ and −/− MEFs were followed during their progression through cell division by live-cell imaging. The cells were monitored by phase microscopy and the length of cytokinesis was calculated from cleavage furrow ingression. E1A/Ras *Hipk2+/+* MEFs underwent an apparently normal cytokinesis in 78.3 ± 36.5 min (n=55), whereas E1A/Ras *Hipk2−/−* MEFs took significantly longer to complete this process (133.7 ± 101.8 min; n=49). In contrast, the time that cells spent in mitosis before anaphase and anaphase duration were not remarkably different between E1A/Ras *Hipk2*+/+ and −/− MEFs (data not shown). Besides the increased length in cytokinesis, E1A/Ras *Hipk2−/−* MEFs displayed marked difficulties in completing cell division, with cells remaining interconnected by intracellular bridges for a long time and with a high percentage of cells that fail cytokinesis (Figures 1D-H and Supplementary Movies S1-S3). Strikingly, 31% of E1A/Ras *Hipk2−/−* MEFs failed cytokinesis ending up as binucleated cells (Figure 1D). These binucleated cells were observed to enter an unhindered mitosis and produce vital progeny (n=8; Figure 1H and Supplementary Movie S3).

### Cytokinesis failure of E1A/Ras *Hipk2−/−* MEFs leads to aneuploidy and CIN

**Figure 2:**
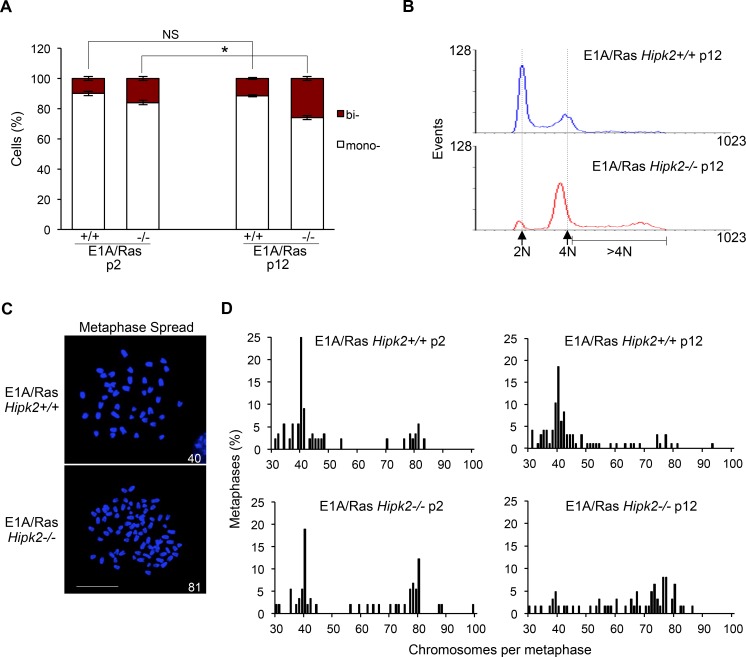
CIN and aneuploidy in E1A/Ras MEFs A, *Hipk2+/+* and *−/−* MEFs were fixed at indicated passages (p) after stable transfection, stained with Hoechst and anti-beta-Tubulin-Cy3 Ab to identify the nuclei and the cytoplasm, respectively. About 1,000 cells per sample were scored for the presence of one or two nuclei/cell and the data are represented as mean ± SD (*P <0.05, Student *t* test). B, DNA content analysis of E1A/Ras MEFs at p12 after stable transfection. Dashed lines outline 2N and 4N DNA content. C, Representative images of Hoechst-stained metaphase spreads of indicated MEFs are shown; scale bar, 10 μm. Chromosome number is reported in each image. D, The percentage of metaphases with the indicated chromosome number is shown; at least 65 metaphases were analyzed for each sample.

To investigate the occurrence of CIN after HIPK2-dependent cytokinesis failure, we measured the frequency of binucleated cells that accumulate during the passages of asynchronously growing MEFs. The morphological evaluation of adherent MEFs was assessed after tubulin immunostaining (Figure 2A). A higher frequency of binucleated cells was observed in the E1A/Ras *Hipk2*−/− MEFs compared with the *Hipk2+*/+ counterparts at early passage after stable transfections. The fraction of binucleated cells increased with passages only in E1A/Ras *Hipk2−/−* MEFs, suggesting that a process of CIN was present after cytokinesis failure due to the hipk2 absence (Figure 2A). At late passages after stable transfection, we also analyzed DNA content of the E1A/Ras MEFs by cytofluorimetric analysis. A strong reduction of the diploid population with a shift towards cells with a double DNA content and a broad population of cells with DNA content >4N, rather than the appearance of a distinct peak of 8N cells, suggest the occurrence of near-tetraploid cells in the E1A/Ras *Hipk2−/−* MEFs (Figure 2B).

To confirm that E1A/Ras MEFs become aneuploid rather than remain tetraploid, we analyzed their karyotypes by chromosomal counts of colcemid-arrested metaphase spreads (Figures 2C-D). E1A and Ras oncogenes are able to induce CIN [33] and in agreement, we detected the presence of tetraploid and near-tetraploid karyotypes (80 ± few chromosomes) in both E1A/Ras *Hipk2+/+* and *−/−* MEFs. However, early passage E1A/Ras *Hipk2−/−* MEFs showed a significant larger accumulation of tetraploid/near tetraploid karyotypes than the *Hipk2+/+* counterparts (Figure 2D). At later passages, the majority of mitoses in the E1A/Ras *Hipk2−/−* MEFs were near tetraploid and a wide distribution of chromosome numbers in the 4N-8N interval was observed, indicative of an ongoing CIN process (Figure 2D). These findings demonstrate the occurrence of CIN by hipk2 deficiency and clearly indicate that oncogene-induced CIN is strongly exacerbated by hipk2 absence. Comparable results were obtained by examining the karyotype of five single-cell clones and three independent polyclonal populations of *Hipk2+/+* and *−/−* MEFs stably-expressing E1A/Ras, indicating that CIN is a specific effect due to HIPK2 status and not to any potential effect deriving from differential E1A/Ras expression in the analyzed populations (data not shown). Moreover, we analyzed also the karyotype of human tumor HeLa cells undergoing cytokinesis failure after HIPK2 transient depletion. Accordingly to near-tetraploidization observed in *Hipk2−/−* MEFs, we observed an increase of the metaphases with near-double chromosome number also in human HeLa HIPK2-depleted cells compared to control cells (Supplementary Figure S1 B-D). Further signs of increased karyotype defects in the E1A/Ras *Hipk2−/−* MEFs were obtained by analyzing the percentage of micronucleated cells (5 ± 0.8% in *Hipk2−/−* MEFs *versus* 1 ± 0.9% in *Hipk2+/+* MEFs), a sign of CIN [34] and by measuring the size of nuclear areas (Supplementary Figure S2A), a parameter that correlate with ploidy [35].

Altogether, these data show that Hipk2 absence leads to accumulation of aneuploidy and CIN.

### E1A/Ras *Hipk2−/−* MEFs are markedly more tumorigenic than E1A/Ras *Hipk2+/+* MEFs and generate highly aneuploid tumors *in vivo*

**Figure 3:**
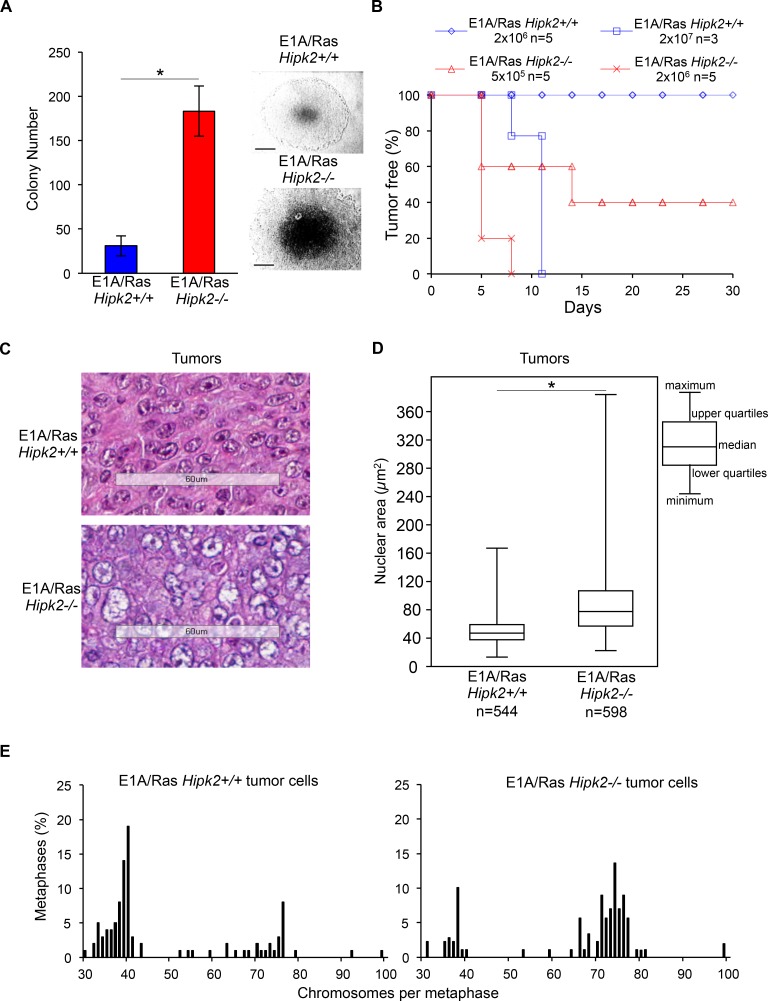
E1A/Ras MEFs tumorigenicity A, Anchorage-independent growth of indicated MEFs was analyzed. The number of colonies obtained by seeding 3 × 10^4^ cells at p2 after stable transfection are presented as mean ± SD. (*P <0.05, Student *t* test). Representative bright-fields of 10 days colonies are shown, right; scale bar, 200 μm. B, Kaplan-Meier tumor free curve is reported for indicated cells concentration. n= mouse number. C, Representative HE staining of indicated tumors; scale bar, 60 μm. D, Morphometrical evaluation of HE-stained sections from three different *Hipk2+/+* and *−/−* tumors was performed. Nuclear area size distribution is reported in box plot graph (*P<0.001, Kruskal-Wallis non-parametric test). E, Metaphase karyotype distribution of indicated tumor-derived cells is shown; at least 90 metaphases were analyzed for each tumor.

To further characterize the phenotype produced by hipk2 absence, we evaluated the tumorigenicity of E1A/Ras *Hipk2+/+* and *−/−* MEFs *in vitro* and *in vivo*. First, we examined the anchorage-independent growth capability by soft agar colony formation assay. We observed that E1A/Ras *Hipk2−/−* MEFs formed more colonies than E1A/Ras *Hipk2+/+* MEFs and that these colonies were characterized by larger dimensions (Figure 3A).

Next, we compared the *in vivo* tumorigenicity of E1A/Ras MEFs by testing their ability in forming tumors in immunocompromised mice. When injected subcutaneously into nude mice (n=5), E1A/Ras *Hipk2−/−* MEFs (2×10^6^) produced rapid and aggressive tumors in all animals within 5 days (Figure 3B and Table 1). In contrast, the same number of E1A/Ras *Hipk2+/+* MEFs was not able to produce detectable tumors during five months of observation. In order to determine the tumorigenic potential of E1A/Ras MEFs more accurately, serial dilution injections were performed. As shown in Figure 3B and Table 1, as little as 1×10^4^ E1A/Ras *Hipk2−/−* MEFs were still able to induce tumors in 1 out of 3 mice in a short period of time (14 days). In contrast, at least 2×10^7^ E1A/Ras *Hipk2+/+* MEFs were required to induce tumors in mice after subcutaneous injection. These findings clearly indicate that E1A/Ras *Hipk2−/−* MEFs are markedly more tumorigenic than their hipk2 proficient counterpart.

**Table 1:** Tumorigenic potential of indicated MEFs *in vivo*

	E1A/Ras Hipk2+/+				E1A/Ras Hipk2−/−			
Injected cell number	2×10^6^	5×10^6^	1×10^7^	2×10^7^	1×10^3^	1×10^4^	5×10^5^	2×10^6^
Tumor incidence	0/5	0/5	0/5	3/3	0/3	1/3	3/5	5/5
Tumor appearance	n.a.	n.a.	n.a.	7	n.a.	13	5	5

Tumor incidence is reported as tumor bearing mice/number of injected mice. Tumor appearance is reported as days post injection. n.a., not applicable.

Impairment of HIPK2 provokes resistance to UV- or doxorubicin-induced cell death and this phenotype is believed to contribute to tumorigenicity [36,37] Indeed, our E1A/Ras *Hipk2−/−* MEFs are more resistant than their *Hipk2+/+* counterpart to doxorubicin-induced cell death (Supplementary Figures S2B and S2C). Thus, in order to assess whether the aneuploidy and CIN we observed in the E1A/Ras *Hipk2−/−* MEFs also contribute to the high tumorigenicity of these cells, we made use of two different experimental approaches. First, we took advantage of a phosphomimetic histone H2B-S14D mutant that, at variance from wild-type H2B, can rescue the cytokinesis failure, in the HIPK2-defective cells [13]. Thus, *Hipk2*−/− primary MEFs were stably transfected with the E1A and Ras oncogenes in combination with wild-type H2B or H2B-S14D (Supplementary Figures S3A). As expected, only the phosphomimetic H2B-S14D mutant was able to rescue the cytokinesis defects (Supplementary Figures S3B-C) When analyzed for the anchorage-independent growth capability, the E1A/Ras *Hipk2−/−* MEFs expressing H2B-S14D showed a strong significant reduction of colony formation compared to E1A/Ras *Hipk2−/−* MEFs expressing wild-type H2B (Supplementary Figures S3D), supporting the idea that cytokinesis failure contribute, at least in part, to the tumorigenicity of the E1A/Ras *Hipk2−/−* MEFs.

Since only 31% of the E1A/Ras *Hipk2−/−* MEFs undergo cytokinesis failure (Figure 1C), we reasoned that if aneuploidy and CIN do not significantly contribute to the tumorigenicity of these MEFs, the karyotype-defective cells would have been counter selected in favor of the cells that succeed in faithful cytokinesis. To experimentally assess this idea, we examined the tumors formed by E1A/Ras *Hipk2+/+* and *−/−* MEFs *in vivo*. Mice were sacrificed and the explanted tumors were processed for histochemical analyses and *in vitro* cell culture. Morphological evaluation of Hematoxylin Eosine (HE)-stained tumor slides showed that both E1A/Ras *Hipk2+/+* and *−/−* MEF-derived tumors were highly malignant sarcomas (Figure 3C). However, when the size of nuclear areas was quantified on a subset of randomly selected tumor regions by using morphometric software, we found that the mean nuclear area of mononucleated *Hipk2−/−* tumor cells was significantly higher than that of *Hipk2+/+* tumor cells (Figure 3D and Supplementary Figure S4). Comparable results were obtained by measuring the mean nuclear area and the mean length of the major nuclear axis by using the Image J software. Together, these data suggest that *Hipk2−/−* tumor cells have a higher DNA content than the *Hipk2+/+* counterpart, supporting the occurrence of an increased ploidy in the absence of hipk2.

To further measure the degree of aneuploidy of the E1A/Ras *Hipk2+/+* and *−/− MEF*-derived tumors, we generated cell lines from the tumors morphometrically analyzed above. Chromosome counts of metaphase spreads showed a clear prevalence of near-tetraploid karyotypes in the E1A/Ras *Hipk2−/−* tumors at opposite with *Hipk2+/+* tumor cells, that were mostly in the diploid range (Figure 3E), suggesting that aneuploid cells are not counter-selected *in vivo*, during tumor formation. Indeed, by comparing the percentages of near-tetraploid metaphases of the E1A/Ras *Hipk2−/−* MEFs before and after *in vivo* passage (compare results in Figures 2D and 3E), an increase of cells with altered karyotype is detectable upon *in vivo* tumor growth, indicating a pro-tumorigenic role for these alterations.

Altogether, these findings demonstrate that hipk2 absence strongly increases tumorigenicity of E1A/Ras-transformed MEFs and support the idea that aneuploidy and CIN contribute to aggressiveness of HIPK2 defective tumors.

### Aneuploidy and CIN caused by Hipk2 absence do not promote transformation

**Figure 4:**
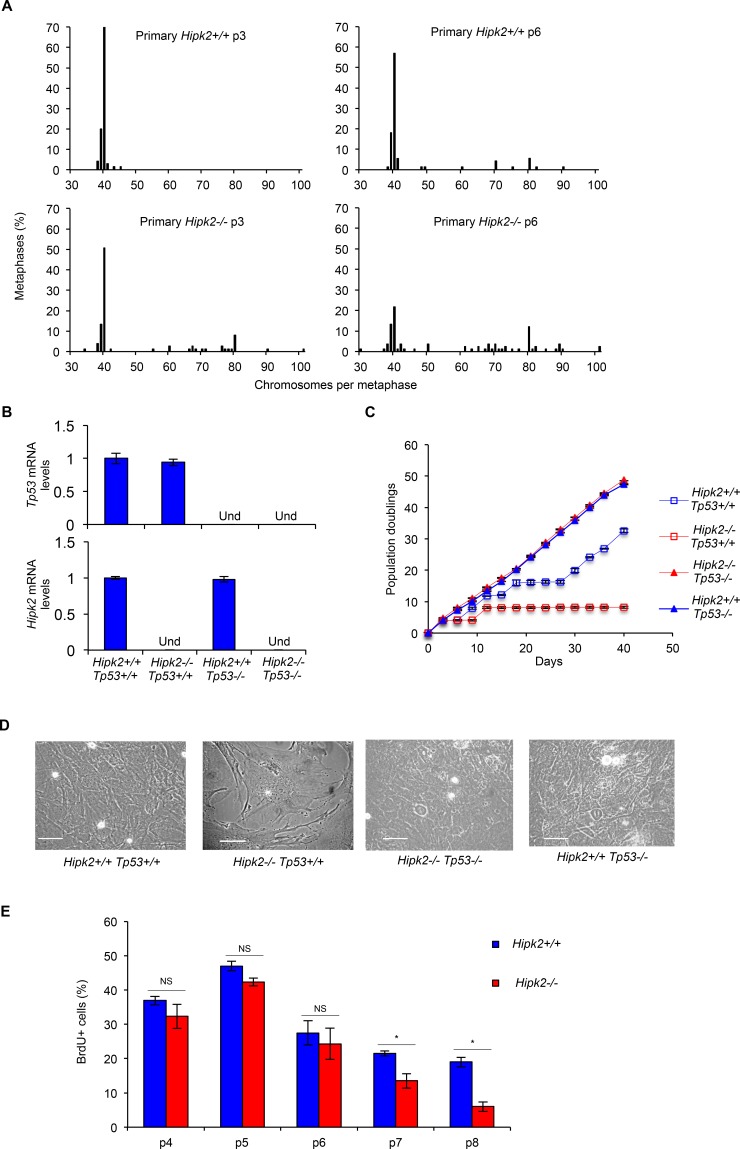
CIN and proliferation in primary MEFs A, MEFs derived from three *Hipk2+/+* and *−/−* embryos from two independent litters were analyzed; the percentage of metaphases with the indicated chromosome number is shown. B, *p53* and *Hipk2* mRNA levels of indicated MEFs were analyzed by quantitative real time RT-PCR at p2 after explantation as control. Relative fold-enrichments were determined by the 2^−ΔΔCt^ method, using Actin as normalizer, and data are represented as mean ± SD. C, Doublings of indicated MEFs were scored and a representative curve of three different experiments performed in triplicate is shown, right. Data are presented as mean ± SD. D, Representative bright-fields of indicated MEFs at p7 after explantation are shown, scale bar, 50 μm. E, MEFs proliferative activity was evaluated as the percentage of BrdU incorporation. BrdU positivity was measured at the indicated p and data presented as mean ± SD (* P= 0.037 at p7 and P= 0.012 at p8, Student *t* test).

We have originally demonstrated that hipk2 absence causes cytokinesis failure and tetraploidization in primary human and mouse fibroblasts [13]. By live-cell imaging, we show here that primary *Hipk2−/−* MEFs have significant longer cytokinesis time and higher percentage of binucleation compared to primary *Hipk2+/+* MEFs (Supplementary Figures S5A-B). Based on the results obtained with the E1A/Ras MEFs, we asked whether the cytokinesis failure observed in the primary MEFs also lead to CIN and promote tumor formation. Thus, we performed chromosomal counts of colcemid-arrested metaphase spreads of primary *Hipk2+/+* and *−/−* MEFs at different passages in culture. As expected, primary *Hipk2+/+* MEFs showed a very stable diploid karyotype (<1% tetraploid cells at p3) with the appearance of a few tetraploid cells at later passages (≅7% tetraploid cells at p6) (Figure 4A). In contrast, *Hipk2−/−* MEFs showed a high percentage of cells with tetraploid and near-tetraploid karyotype from the early passages (≅25% tetraploid/near-tetraploid cells at p3) that further increased at later passages (≅45% tetraploid/near-tetraploid cells at p6) (Figure 4A). These observations suggest that the tetraploidization events occurring in *Hipk2−/−* MEFs due to cytokinesis failure are followed by other cell divisions with missegregation of one or a few chromosomes that lead to CIN and aneuploidy around the tetraploid state. In addition, some near-tetraploid progeny might also derive by near-diploid cells that fail cytokinesis in the absence of Hipk2.

Next, we evaluated whether aneuploidy and CIN are sufficient to induce transformation in primary MEFs. We first compared population doublings and spontaneous immortalization in primary *Hipk2*+/+ and −/− MEFs by routinely passaging the cells by the 3T3 protocol. As expected, *Hipk2*+/+ MEFs proliferate and, after a crisis, resume proliferation (Figures 4C-D), becoming immortal. In contrast, primary *Hipk2−/−* MEFs, after the first passages in which accumulate karyotype defects (Figure 4A), stop proliferating and do not spontaneously immortalize (Figures 4C-D). This different behavior was reproducibly seen in littermate-paired MEFs derived from three independent litters and was confirmed by bromodeoxyuridine (BrdU) incorporation analyses (Figure 4E).

To evaluate whether the *Hipk2−/−* MEFs stop proliferating because of a tetraploid G1 arrest induced by tumor suppressive mechanism such as p53 activation [20], we analyzed the effect of hipk2 absence in p53-null background. We observed that *Hipk2−/− Tp53−/−* MEFs, despite the presence of CIN, proliferate and spontaneously immortalize, as well as *Hipk2+/+ p53−/−* MEFs, suggesting that p53 inactivation leads to the acquisition of tolerance to the CIN induced by hipk2 absence (Figures 4B-D).

Overall, these observations indicate that hipk2 absence leads to tetraploidy associated with aneuploidy and CIN in primary MEFs and suggest that these events, despite an initial proliferation of tetraploid/near-tetraploid cells, inhibit rather than facilitate tumor promotion. In agreement with these findings, we observed that hipk2 absence is not sufficient to trigger transformation of primary MEFs by expressing a single oncogene, such as Ras or E1A (Supplementary Figure S6). In addition, we observed that *Hipk2−/− Tp53−/−* MEFs, such as *Hipk2+/+ p53−/−* MEFs, do not show anchorage-independent growth capability, suggesting that hipk2 absence is not sufficient to induce transformation even in primary MEFs lacking p53.

Together with the data of the E1A/Ras transformed MEFs, the results we obtained with the primary MEFs support a role for hipk2 inactivation in tumor progression rather than in tumor promotion.

### Reduced HIPK2 expression correlates with high tumor and nuclear grade in pancreatic adenocarcinoma

To verify whether the relationship between hipk2 absence and CIN defined in MEFs can occur in human cancers, we evaluated the HIPK2 expression in tissue microarrays (TMAs) of pancreatic cancers in which tetraploidization due to cytokinesis failure precedes an aneuploid state characterized by high incidence of near-tetraploid karyotypes [29-32].

**Figure 5:**
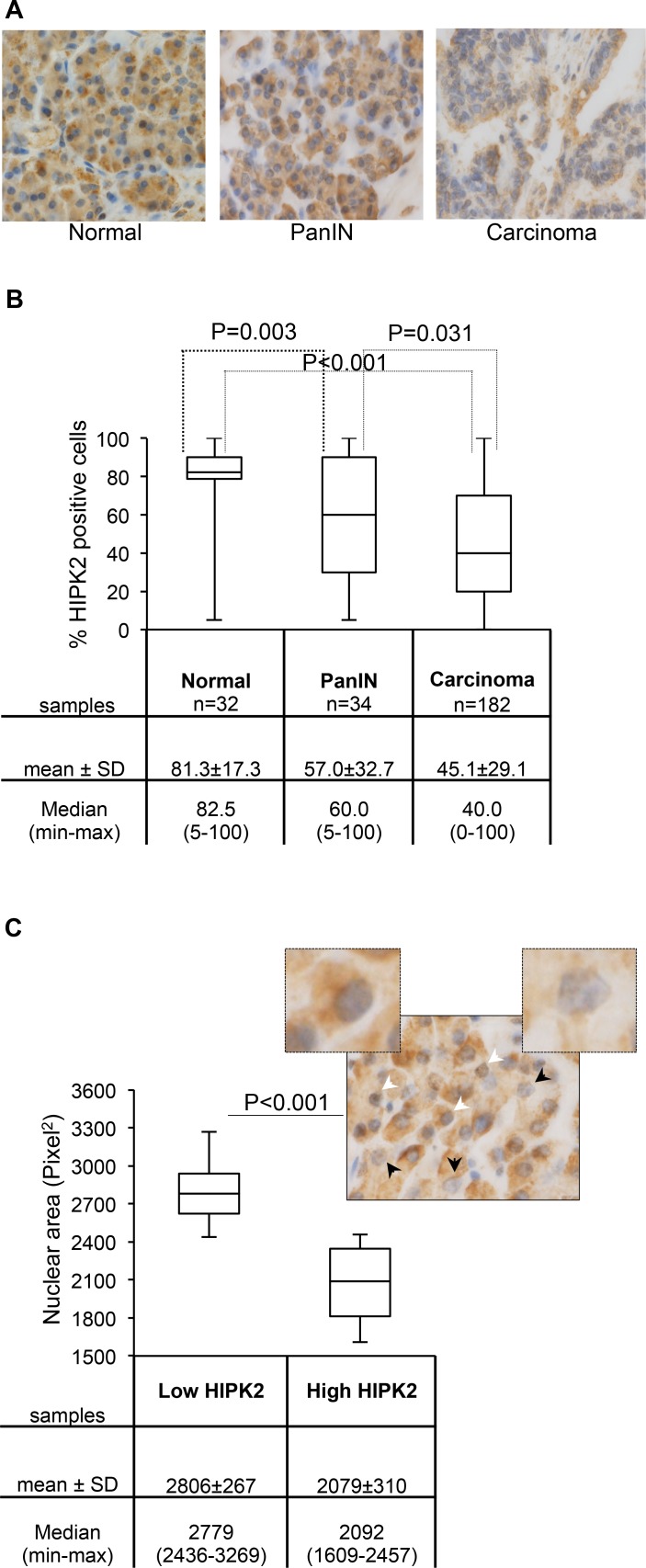
HIPK2 expression during pancreatic malignant progression A, Representative images of HIPK2 immunostaining in normal pancreas, PanIN-3, and adenocarcinoma samples. B, The percentage of HIPK2 positive cells observed in each indicated subset of samples is reported and P values for the Wilcoxon test are shown. C, Nuclear area size distribution of low and high HIPK2-expressing cells in PanIN-3 samples (n=12) was evaluated and reported; for each case 400 tumor nuclei were measured and P value for the Student *t* test is shown. Low and high HIPK2-expressing cells show 2.3 fold of difference in the intensity of the staining. A representative PanIN-3 image in which the black arrows indicate low HIPK2 expressing cells and the white arrows indicate high HIPK2 expressing cells is reported and 5X magnification of indicated cells is shown.

Immunohistochemical analyses were performed by using anti-HIPK2 specific antibody (Ab) [11] in TMAs that included normal pancreatic tissue, PanIN-3, and invasive ductal adenocarcinomas [38]. As shown in Figures 5A and 5B, we found that the percentage of HIPK2 positive cells, irrespective of the intensity of the staining, was significantly reduced in PanIN-3 and in pancreatic adenocarcinoma compared to normal tissue. It is worthy to note that we also observed a general decrease in the intensity of HIPK2 staining by comparing samples of normal *versus* PanIN-3 and PanIN-3 *versus* adenocarcinoma (Figure 5A and data not shown). A further analysis of the latter finding in the PanIN-3 samples highlighted a relationship between HIPK2 intensity and the shape and the size of nuclei. In particular, to quantify this aspect, cells were divided into high and low HIPK2 expressing cells and nuclei were analyzed by measuring the area and the length of the major axis. Binucleated cells were not considered for these analyses. We found a highly significant correlation between low HIPK2-expressing cells and ample, pleomorphic, nuclei and between high HIPK2-expressing cells and small, regularly shaped nuclei (Figure 5C and data not shown). A representative PanIN-3 image in which the black arrows indicate low HIPK2 expressing cells and the white arrows indicate high HIPK2 expressing cells is reported and 5X magnification of indicated cells is shown.

These results show, in pancreatic cancer, an association between HIPK2 reduction, and high tumor and nuclear grades. Although this association needs to be further investigated, it opens up to the possibility that HIPK2 reduction/inactivation might contribute to tetraploidization and CIN in this type of cancer.

## DISCUSSION

Unscheduled tetraploid cell proliferation can lead to CIN and ultimately to cancer [16, 17,22]. In this study, we observed that cytokinesis failure and tetraploidization caused by HIPK2 absence evolve rapidly in high levels of aneuploidy and CIN. As a consequence, we observed that accumulation of these defects antagonizes cell proliferation and spontaneous immortalization of primary MEFs whereas it associates with increased tumorigenicity in E1A/Ras transformed MEFs. Hipk2-null MEFs proliferation and immortalization can occur in the background of non functional p53 tumor suppressor protein, according to data showing that tetraploids expressing wild-type p53 fail to propagate [16]. However, hipk2 absence does not induce transformation in a p53-null context in our experimental system. These findings suggest that HIPK2 absence is not sufficient for tumor promotion, but rather favors tumor progression destabilizing the genome, as reflected by chromosomal abnormalities found in hipk2-null cells. These data are in agreement with observations reporting that hipk2 absence does not induce spontaneous tumors in mice but predisposes to tumor formation upon chemical or physical induction. Altogether, these findings support a new model where HIPK2 acts as a caretaker gene, whose absence causes CIN, which triggers further alterations in the presence of oncogenic stress and ultimately accelerate tumor progression toward a higher malignant state. It was proposed that more potent CIN genes are those that possess multiple tumor suppressive activities that are simultaneously perturbed [26]. Although HIPK2 does not act directly as a tetraploid checkpoint protein, our findings, when combined with the role of HIPK2 in DNA damage checkpoint control, indicate that HIPK2 might be important for safeguarding the genome, not only by participating in the DNA damage responses but also by controlling genome stability and preventing tetraploidy.

It has been reported that E1A/Ras transformed p53-null MEFs display increased CIN caused by increased ROS production [33]. Recently, HIPK2 was shown to be required also for ROS-induced cell death [39], but whether this is another reason for the increased CIN in hipk2-null MEFs has to be further investigated.

HIPK2 inactivation/dysfunction has been observed in different types of human tumors and several evidences support the notion that the dosage of this tumor suppressor might be relevant. In particular, it has been reported that HIPK2 loss-of-heterozygosity occurs frequently in radiation-induced mouse lymphomas and in human thyroid carcinomas, indicating that loss of a single allele is sufficient to impact tumor susceptibility, at least in some contexts [5, 40]. Rare mutations or amplification of the *HIPK2* gene in some human cancers have also been reported [41, 42]. However, data are sparse and the HIPK2 role in tumor formation and/or progression is still unclear in a scenario that appears complex and heterogeneous. Thus, it is important to identify *HIPK2* as one of the CIN genes with different tumor-protective molecular activities, because it might facilitate to decipher the basis of heterogeneity of HIPK2 dysfunctions in different tissues and subsets of cancers.

Pancreatic adenocarcinomas belong to tumors in which relevant oncosuppressor pathways are believed to be inactivated before the emergence of tetraploidization [29-32, 43]. This characteristic makes them interesting to look for HIPK2 dysfunctions. In this study, we found for the first time that HIPK2 was under-expressed in PanIN-3 and in pancreatic adenocarcinoma in contrast to the high expression levels observed in normal pancreas. This novel finding is consistent with the function of HIPK2 as a tumor suppressor during pancreatic malignant progression. However, the mechanisms underlying HIPK2 inactivation in this type of cancer remains to be revealed. Interestingly, the nuclei of the PanIN-3 cells are reported to exhibit large pleiomorphism and considerable changes in the nuclear area that often correlate with altered DNA content [44-47]. Molecular alterations occurring during PanIN1-3 progression have been identified and the inactivation of relevant oncosuppressor pathways such as those of pRb and p53 has been frequently observed before the tetraploidization stimulus at PanIN-3 [32, 44]. Our observations indicate that low HIPK2 expressing cells are greater in nuclear size than high HIPK2 expressing cells in the PanIN-3 lesions. Although this correlation needs further study, it opens a scenario in which HIPK2 reduction might correlate with higher DNA ploidy. Thus, these data might suggest that HIPK2 dysfunction can be one of the causes of cytokinesis failure leading to tetraploidization in the precursor lesions during pancreas tumor progression. Furthermore, the HIPK2 expression reduction in PanIN lesions would be a potential marker for the early detection of pancreatic neoplasia. Recent molecular studies show the importance of these lesions as precursors to invasive pancreatic cancer and highlight the relevance of PanINs in cancer treatment, as their early detection would be helpful in treating them before an invasive cancer develops.

## METHODS

### Cells and culture conditions

Primary MEFs explantation was performed as previously described [13]. Primary MEFs were cultured in DMEM with high-glucose, supplemented with 20% heat-inactivated FBS (HyClone, Thermo Scientific, Lafayette, CO, USA). *Tp53* heterozygote mice were kindly provided by Prof. P. Di Fiore; 48). Human cervical adenocarcinoma HeLa cells and E1A/Ras MEFs were cultured in DMEM with low-glucose, supplemented with 10% heat-inactivated FBS (Life Technologies, Carlsbad, CA, USA). To determine population doublings a 3T3 subculture schedule was performed by plating 3 × 10^5^ cells per 60 mm dish in triplicate.

### BrdU incorporation assay

Cells were incubated in the presence of 20 μM BrdU (Sigma, Saint-Louis, MI, USA) for 16 h, fixed in methanol/acetone 1:1 and subjected to immunofluorescence with anti-BrdU Ab (1:100 dilution; DAKO, Glostrup, Denmark) and FITC-conjugated secondary Ab (1:200 dilution; Jackson Labs, Bar Harbor, ME, USA). Nuclei were counterstained with 1 μg/ml Hoechst 33258 dye (Sigma). At least 500 cells per sample were counted.

### Soft agar colony formation assay

Cells were suspended in 0.6% Difco agar noble (Becton Dickinson, Sparks, MD, USA) in growth medium, plated on 60 mm dish containing a solidified bottom layer (1.2% agar in growth medium) and incubated.

### RNA interference, RNA extraction and quantitative real-time RT-PCR

RNA interference was obtained by HIPK2-specific stealth RNAi sequences (a mix of three different sequences in combination) and by universal negative control stealth RNAi, the Negative Medium GC Duplexes (Life Technologies) as reported (13). RNA extraction and quantitative real-time RT-PCR were performed as in ref. 11. The following primers were used: Forward Hipk2 5′-AGGAAGAGTAAGCAGCACCAG-3′; Reverse Hipk2 5′-TGCTGATGGTGATGACACTGA-3′; Forward Actin 5′-CGATGCCCTGAGGCTCTTT-3′; Reverse Actin 5′-TAGTTTCATGGATGCCACAGGAT-3′; Forward p53 5′-CCTCTGAGCCAGGAGACATTTTC-3′; Reverse p53 5′-AAGCCCAGGTGGAAGCCATAGTTG-3′; Each target amplification was performed in duplicate on two different RNA preparations.

### Tumorigenicity assay and tumors morphological analyses

Cells were suspended in PBS and injected subcutaneously, within the interscapular region, in 6 week-old female nude mice. To avoid *in vitro* selection, cells were expanded minimally before injection; MEFs at passage 3 after stable transfection were injected. Mice were monitored for tumor appearance/growth three times a week. Tumor growth was followed by caliper measurements and tumor volume (TV) estimated by the formula: TV = a × b^2^/2, where a and b are tumor length and width, respectively. Tumor bearing mice were sacrificed when TV was up to 2 cm^3^. Excised tumors were fixed in 4% PBS-buffered formalin. After conventional histological preparation 3 μm thick sections were stained with HE for microscopic morphological evaluation. Morphometric analyses were performed on contiguous sections by using ImageJ software (National Institutes of Health, Bethesda, MD, USA) or by automated analysis (Aperio Scan Scope XT/CS). Randomly selected tumor fields were considered.

All the procedures involving animals and their care were conformed to the relevant regulatory standards in accordance with the Italian legislation.

### Expression vectors and transfection

The following plasmids were employed: E1A12S- and Harvey-Ras- expressing vectors (kindly provided by Dr. G. Piaggio; [49]), EGFP-expressing vector (pEGFP-c2; Clontech, Mountain View, CA, USA) and pBabepuro (vector expressing the puromycin-resistance marker), GFP-H2B and GFP-H2BS14D (carrying blastictidine-resistance marker; [13]). Cells were transfected by using Lipofectamine LTX and Plus reagent (Life Technologies) and selected in 2 μg/mL puromycin (Sigma) and/or in 3 μg/mL blasticidine (Sigma).

### Metaphase spreads and flow cytometry analysis

Actively proliferating cells were treated with 100 ng/ml demecolcine solution (Sigma) for 3 h. Cells were trypsinized, washed with PBS, hypotonically treated with 75mM KCI and fixed in methanol/acetic acid 3:1. After two fixative changes, cells were dropped onto cold slides and stained with Hoechst 33258. For flow cytometry analysis active proliferating cells were permeabilized in PBS/0.1% Triton X, stained with propidium iodide (Sigma) and analyzed by EPICS XL (Beckman Coulter, Brea, CA, USA).

### TMAs

TMAs data validation and immunoistochemical evaluation were performed as previously described [38]. The Ab used for HIPK2 immunostaining was described in ref. 11. Each sample was scored and analyzed independently by two pathologists. For morphometric analyses, HIPK2 staining intensity was graded as absent (0), weak (1+), intermediate (2+), or strong (3+) and the cells were divided into two groups, high (intermediate/strong) and low (absent/weak) HIPK2 expressing cells.

### Live-cell imaging

Cells seeded in μ-slides 8-well (80826, Ibidi, Munchen, Germany) were observed under a Nikon Eclipse Ti inverted microscope using a 40x objective. During the whole observation, cells were kept in a microscope stage incubator (Basic WJ, Okolab) at 37°C and 5% CO_2_. DIC images were acquired over a 24 hours period by using a DS-Qi1Mc camera. Image and video processing was performed with NIS-Elements AR 3.22.

### Western blotting

Total Cell Extracts (TCEs) were prepared and resolved as previously described [13]. The following Abs were employed: anti-Ras (OP40, 1:500 diluition; Calbiochem, Cambridge, UK), anti-E1A (M58 clone, 1:500 diluition; BD Biosciences, San Jose, CA, USA), anti-GAPDH (1:1000 diluition; Santa Cruz Biotechnology, Santa Cruz, CA, USA), anti-HIPK2 (kindly provided by Dr. L. Shmidtz); anti-GFP (1:500 diluition; Santa Cruz Biotechnology, Santa Cruz, CA, USA); anti-alpha-tubulin and anti-Actin (1:1000 diluition; Immunological Science, Rome, Italy); anti-HRP-conjugated goat anti-mouse and anti-rabbit (Bio-Rad Laboratories, Hercules, CA, USA). Immunoreactivity was determined using the ECL-chemiluminescence reaction (Amersham, Piscataway, NJ, USA) following the manufacturer's instructions.

### Immunofluorescence

Cells were seeded onto poly-L-lysine coated coverslips, fixed in 2% formaldehyde, washed in PBS, permeabilized in 0.25% Triton X-100 in PBS, and then blocked in 5% bovine serum albumin in PBS before anti-beta-Tubulin-Cy3 (1:500 dilution; Sigma) was applied. Nuclei were counterstained with Hoechst 33528.

### Statistical analysis

Significant changes were assessed by using Student's t test. P values <0.05 were considered significant. Normality tests were performed on measures of the nuclear area in murine explanted tumors. Since data did not follow a normal distribution, they were analyzed using Kruskal-Wallis non-parametric test and the statistical analysis was performed with the PAST free data analysis package. TMA statistics were calculated using Wilcoxon rank sum test and Cox regression analysis.

## SUPPLEMENTARY MATERIALS, FIGURES AND MOVIES








