# Supplementary material for: HIPK2 deficiency causes chromosomal instability by cytokinesis failure and increases tumorigenicity
Source: Oncotarget. 2015 Mar 14;6(12):10320–34. doi: 10.18632/oncotarget.3583 (PMC4496358; doi:10.18632/oncotarget.3583)
Supplement: Supplementary file 1 [file oncotarget-06-10320-s001.pdf]

HIPK2 deficiency causes chromosomal instability by cytokinesis failure and increases tumorigenicity

Supplementary Material

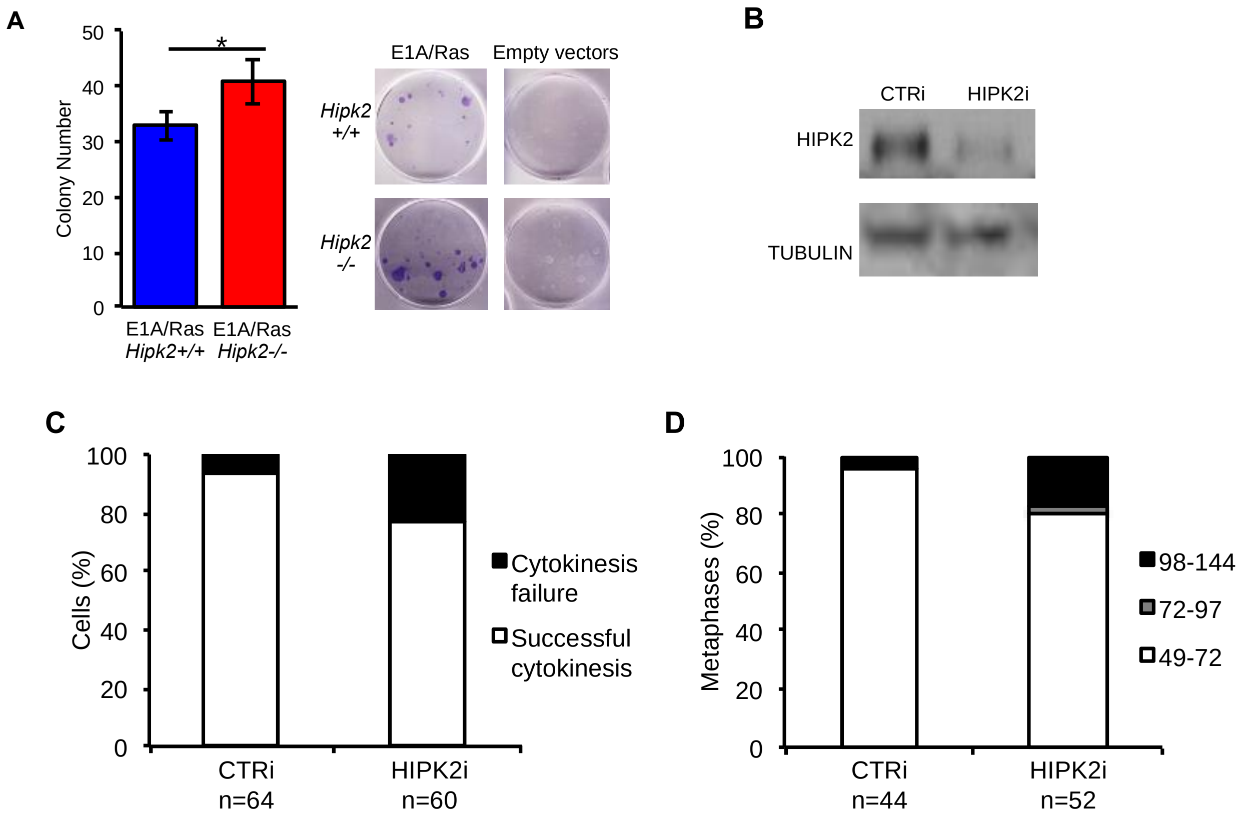

Supplementary Figure S1: hipk2 depleted cells show significant difference in E1A/Ras colony number obtained after selection and in chromosome number. A, Primary *Hipk2*+/+ and -/- MEFs were stably transfected at passage 3 after explantation with the indicated vectors. Transfection efficiency was consistently 11% for the *Hipk2*+/+ and 8% for the *Hipk2*-/- MEFs. The number of E1A/Ras stable-expressing colonies obtained after selection was counted. The experiments were performed in quadruplicate and data relative to transfection efficiency are represented as mean ± SD, left; (* $P<0.05$, Student *t* test). Representative crystal-violet stained plates are shown, right. B-D, HeLa cells were transfected with 40nM of HIPK2 specific stealth siRNA mix (HIPK2i) or universal negative control stealth siRNA mix (CTRi) and analyzed 5 days after transfection. HIPK2 protein levels by WB are shown in B, cytokinesis outcome was monitored by time lapse microscopy and reported in C, the percentage of metaphases with the indicated chromosome number is shown in D.

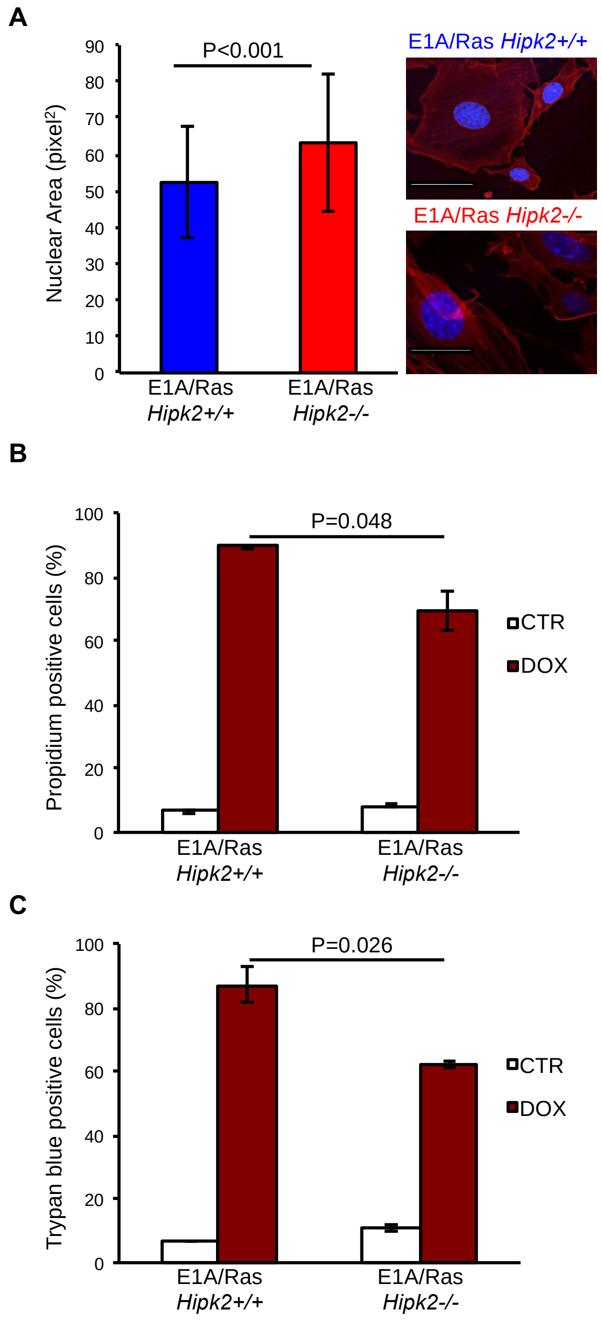

Supplementary Figure S2: E1A/Ras *Hipk2+/+* and -/- MEFs show significant difference in nuclear size and in response to doxorubicin treatment. A, Nuclear area of E1A/Ras *Hipk2+/+* and -/- mononucleated MEFs stained with anti-beta-Tubulin-Cy3 (red) and Hoechst (blue) was measured and reported as mean ± SD; for each case 400 nuclei were measured and P value for the Student *t* test is shown (left). Representative immunostainings of indicated MEFs are shown (right); scale bar, 10μm. B-C, Indicated MEFs were treated with 1 μm doxorubicin (Sigma). Cell viability was assessed by using ADAM-MC automatic cell counting system (Bio Digital) in B and by trypan blue exclusion in C 24 h after treatment. CTR, control untreated cells; DOX, doxorubicin-treated cells. P values for the Student *t* test are shown

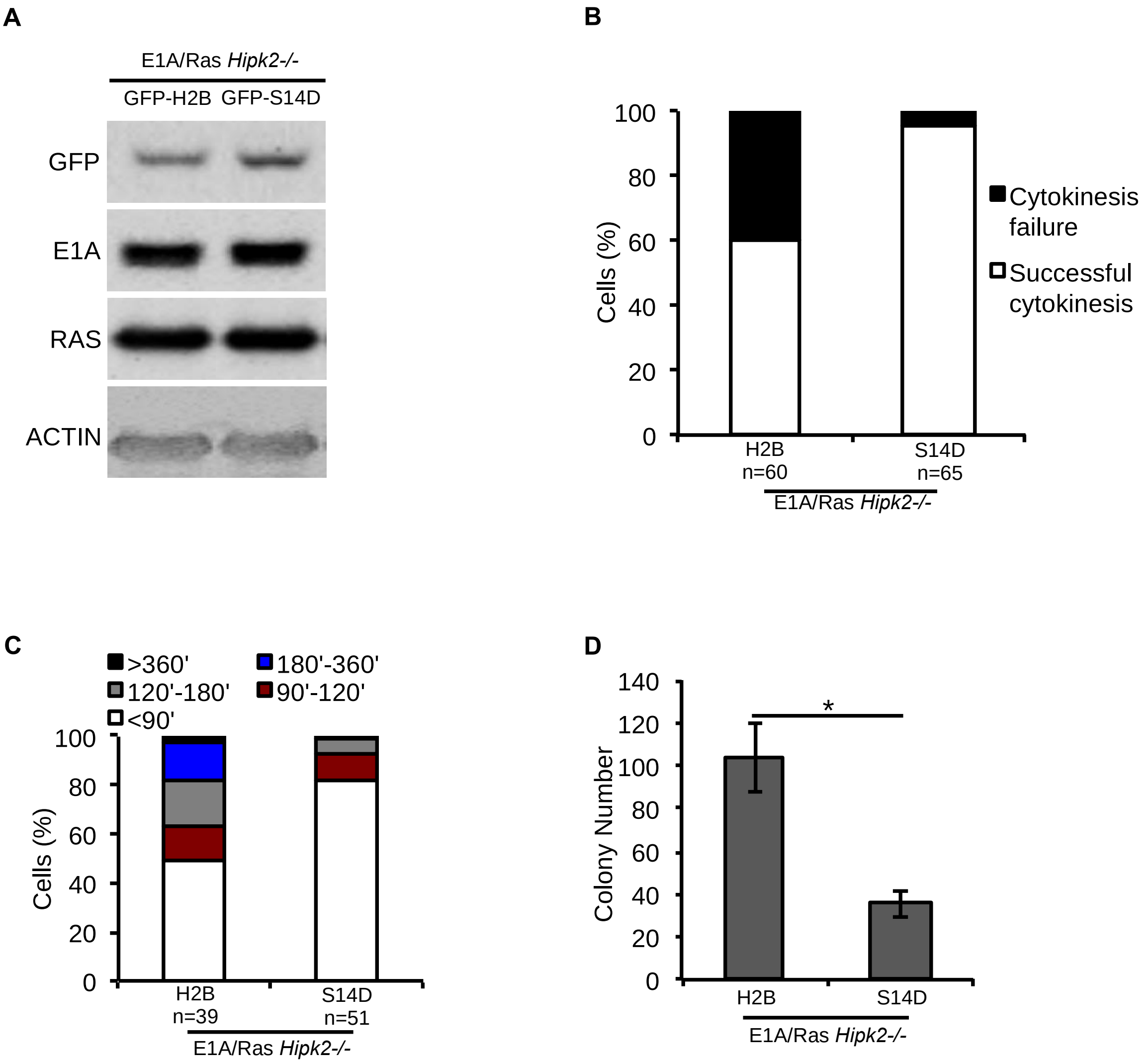

Supplementary Figure S3: Phosphomimetic H2BS14D expression rescue cytokinesis failure and reduce *in vitro* tumorigenicity of E1A/Ras *HIPK2-/-* cells. A-D, primary *Hipk2-/-* MEFs were stably transfected at passage 3 after explantation with E1A and Ras expression vectors in combination with a vector expressing GFP-H2B or GFP-H2B-S14D. A, TCEs from stable polyclonal populations were analyzed by WB to verify expression levels of exogenous proteins by using indicated Abs. ACTIN expression was used as loading control. B-C, Asynchronous polyclonal populations were analyzed at passage 2 after stable transfection by live-cell imaging as in the Figure 1 D-E. The percentage of mononucleated cells with the indicated outcome is reported in B. Cytokinesis time was evaluated for each mononucleated cell successfully completing the cell division and the percentage of cells with the indicated cytokinesis time is reported in C. D, Anchorage-independent growth was analyzed. The soft-agar colony number obtained by seeding 3×10^4 cells at passage 2 after stable transfection are presented as mean ± SD (*P <0.05, Student *t* test).

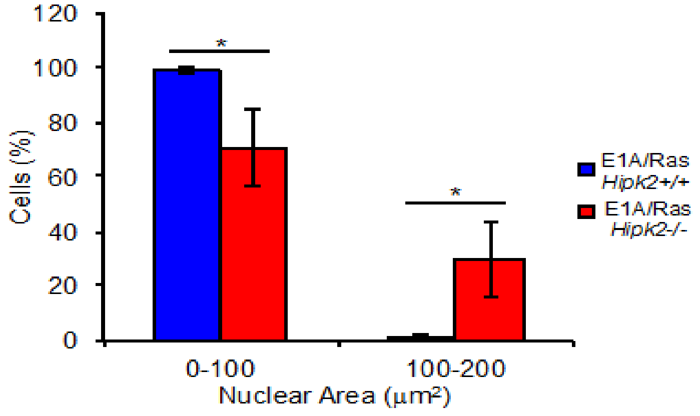

Supplementary Figure S4: Thirty percent of E1A/Ras *Hipk2-/-* derived tumors show larger nuclei than E1A/Ras *Hipk2+/+* derived tumors. Data from morphometrical evaluation showed in Figure 3D were used to calculate the percentage of cells with the indicated nuclear area. Data are presented as mean ± SD (* $P<0.05$, Student *t* test).

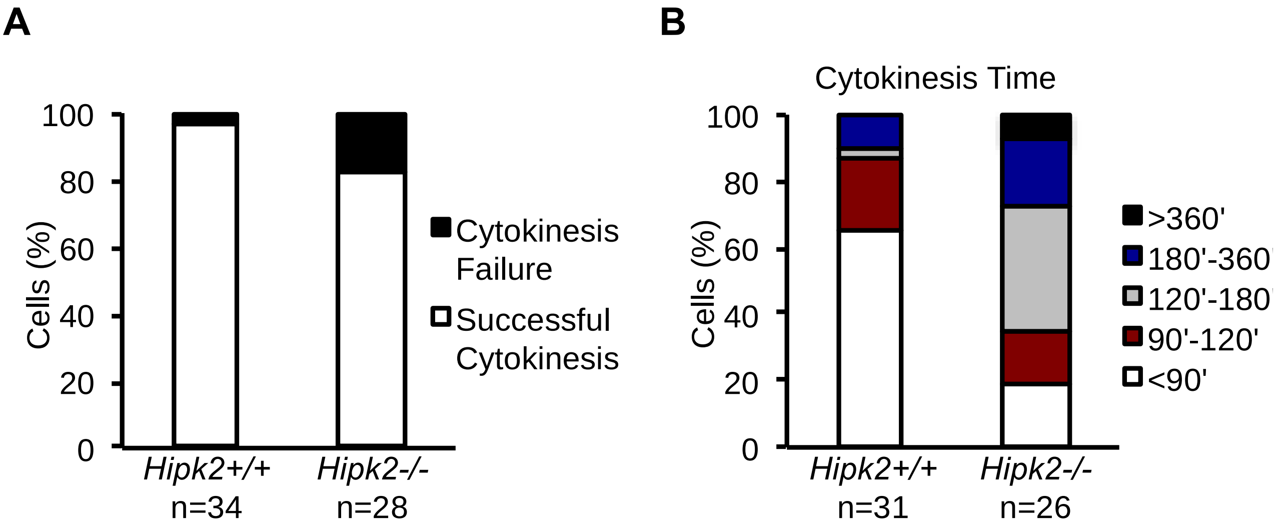

Supplementary Figure S5: Primary *Hipk2-/-* MEFs show cytokinesis failure and longer cytokinesis time than primary *Hipk2+/+* MEFs. A-B, Asynchronous primary MEFs were analyzed by live-cell imaging at passage 4 after explantation. Cells were monitored by phase microscopy and the length of cytokinesis was calculated from cleavage furrow ingression as in Figure 1D-E. The percentage of mononucleated cells with the indicated outcome is reported in A. Cytokinesis time was evaluated for each mononucleated cell successfully completing the cell division and the percentage of mononucleated cells with the indicated cytokinesis time is reported B.

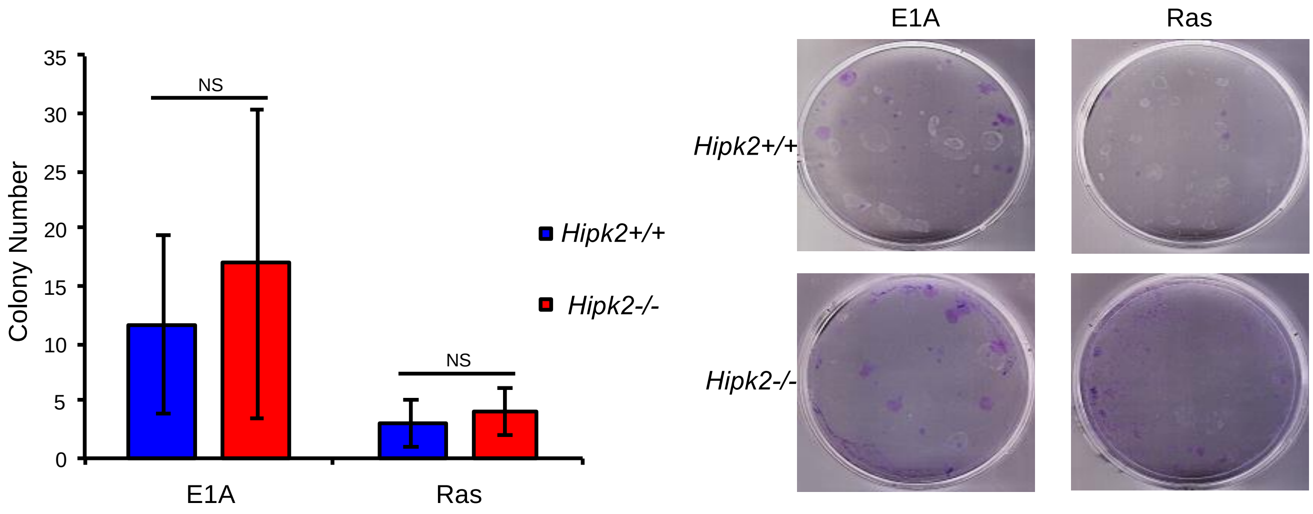

Supplementary Figure S6: Some E1A- or Ras-expressing colonies were obtained by stably transfecting E1A- or Ras- expressing vectors, however these colonies could not be fully established. Primary *Hipk2+/+* and -/- MEFs, at passage 3 after explantation, were stably transfected with the indicated vectors. Number of colonies obtained after 10 days of selection were counted. No colonies were obtained by transfecting empty control vectors. The experiments were performed in triplicate and data are represented as mean ± SD (left); No significant differences between *Hipk2+/+* and -/- populations were observed. Representative crystal-violet stained plates are shown, right. E1A- and Ras-expressing single cell-derived clones or stable polyclonal populations could not be established. Primary MEFs require the presence of two cooperating oncogenes such as adenoviral E1A along with Ras for transformation [50]. The expression of a single oncogene is not sufficient for *Hipk2* null MEFs transformation.

Legend to the Supplementary Movies

Supplementary Movie S1. Time-lapse movie of asynchronous E1A/Ras *Hipk2+/+* MEFs related to Figure 1. The display rate is one frame every 250 millisecond. Still images of this video are shown in Figure 1F.

Supplementary Movie S2. Time-lapse movie of asynchronous E1A/Ras *Hipk2-/-* MEFs Related to Figure 1. The display rate is one frame every 250 millisecond. Still images of this video are shown in Figure 1G.

Supplementary Movie S3. Time-lapse movie of asynchronous E1A/Ras *Hipk2-/-* MEFs related to Figure 1. The display rate is one frame every 250 millisecond. Still images of this video are shown in Figure 1H.
